# Upcycling of Grape Pomace from Malbec, Merlot, Syrah and Grenache: Varietal Effects on Anthocyanin Extract Properties and Performance in Semi-Solid Topical Formulations

**DOI:** 10.3390/foods15091466

**Published:** 2026-04-22

**Authors:** Antonia L. Cruz-Diaz, Valentina V. General, Daniela Orellana, Angie V. Caicedo-Paz, Cassamo U. Mussagy

**Affiliations:** Laboratorio de Desarrollo de Bioprocesos Sostenibles (Labisost), Escuela de Agronomía, Facultad de Ciencias Agronómicas y de los Alimentos, Pontificia Universidad Católica de Valparaíso, Casilla 4-D, Quillota 2260000, Chile; antonia.cruz.d@mail.pucv.cl (A.L.C.-D.); valentina.vasquez.g@mail.pucv.cl (V.V.G.); daniela.orellana.m@mail.pucv.cl (D.O.); angie.caicedo@udea.edu.co (A.V.C.-P.)

**Keywords:** circular economy, pH-responsive pigments, antioxidant activity, extract stability, topical applications

## Abstract

Grape pomace represents a widely available agro-industrial by-product in Chile with considerable potential for valorization within circular economy frameworks; however, its functionality as a cosmetic ingredient depends on both grape cultivar and processing strategy. In this study, the direct incorporation of solid grape pomace residues into cream formulations was first evaluated, revealing limitations related to color control, homogeneity, and sensory performance. Subsequently, the influence of varietal origin (Malbec, Merlot, Syrah, and Grenache) on the extraction, stability, color behavior, and functional performance of anthocyanin-rich extracts was investigated for cosmetic applications. pH-standardized color analysis revealed statistically significant (*p* < 0.05) varietal differences, with Malbec extracts showing superior chromatic stability under acidic and near-neutral conditions, exhibiting lower reduction in a* values across the pH range compared to other varieties. In contrast, Syrah, Grenache, and Merlot showed a more pronounced decrease in red chromaticity, indicating higher sensitivity to pH-induced structural transformations. Although Merlot and Syrah exhibited higher ABTS antioxidant activity, Malbec presented the highest total phenolic content and the most balanced functional profile when considering both stability and color retention. Incorporation of anthocyanin-rich extracts into cosmetic cream formulations demonstrated that a 4.5% (m/v) loading ensured a skin-compatible pH (4.5–5.5), with Malbec-based creams exhibiting superior color stability and formulation performance over time. These findings demonstrate that grape pomace valorization requires variety-specific evaluation and identify extraction as a key enabling step for the development of sustainable, bio-based color-functional cosmetic ingredients.

## 1. Introduction

Chile is one of the world’s leading wine-exporting countries and a recognized reference for sustainable wine production [1,2]. However, large-scale vinification generates significant volumes of solid residues, among which grape pomace is the most abundant and underutilized by-product of the wine industry [3]. Composed mainly of skins, seeds, and residual pulp, grape pomace can represent up to 25–30% of the total processed grape mass worldwide [4]. In wine-producing countries (*viz*., Chile), the seasonal accumulation of this residue poses environmental and logistical challenges, particularly when management practices are limited to landfilling or low-value applications [5]. From a cleaner production perspective, grape pomace should therefore be viewed not merely as a waste stream, but as a strategically important secondary raw material that can be reintegrated into value chains through sustainable upcycling strategies [6,7,8,9].

The valorization of agro-industrial residues is a core pillar of circular economy frameworks, aiming to decouple economic growth from resource depletion while minimizing environmental impacts [10,11,12,13]. Within this framework, grape pomace has emerged as a valuable renewable source of polyphenolic compounds, including phenolic acids, flavonoids and anthocyanins, which are associated with a wide range of biological activities, such as antimicrobial, anti-inflammatory, antioxidant, anticancer, and cardioprotective effects [14]. Among these compounds, anthocyanins stand out due to their intense coloration, antioxidant capacity, and multifunctional properties, which make them suitable candidates for replacing synthetic colorants in food, cosmetic, and personal care products [15,16,17]. The substitution of petrochemical-derived dyes with bio-based alternatives aligns directly with cleaner production goals by reducing fossil resource dependency and improving product environmental profiles [18]. Despite their potential, the industrial implementation of anthocyanin-rich extracts remains limited by stability constraints [15,19]. Anthocyanins are highly sensitive to pH, temperature, light exposure, and oxidative environments, which can lead to rapid degradation and color loss during processing and storage [20]. Consequently, extraction and stabilization strategies must be designed not only to maximize natural pigment recovery but also to ensure functional performance throughout the product life cycle [21,22,23]. Indeed, cleaner production principles emphasize the need for mild operating conditions, energy-efficient processes, and the use of safe, biocompatible, and recyclable and low-cost solvents, positioning hydroalcoholic and acidified aqueous systems as particularly attractive extraction media [5,24]. Beyond process parameters, raw material variability critically affects the performance and reproducibility of bio-based products, with grape pomace anthocyanin composition differing among cultivars and influencing color and stability, Merlot being dominated by malvidin-3-glucoside and peonidin-3-glucoside with few acylated forms, Malbec showing a broader profile including high malvidin-3-glucoside, delphinidin-3-glucoside and acylated derivatives, Syrah rich in malvidin-3-glucoside, peonidin-3-glucoside and acylated anthocyanins enhancing pigment stability, and Grenache exhibiting a simpler profile mainly of malvidin-3-glucoside and cyanidin-3-glucoside with lower total anthocyanins and fewer acylated forms [25,26]. Note that these varietal differences can significantly influence extraction efficiency, chromaticity, pH responsiveness, antioxidant activity, and stability. However, many valorization studies treat grape pomace as a homogeneous feedstock, overlooking varietal effects and limiting the transferability of results to industrial settings. For cosmetic and personal care applications for example, additional performance requirements must be met, including color stability at near-physiological pH (4.5–5.5), compatibility with formulation matrices, and controlled color variation over storage time [27]. Failure to account for these constraints can compromise product quality, consumer acceptance, and shelf life, undermining the sustainability benefits of bio-based ingredients. Therefore, an integrated evaluation that links extraction efficiency, varietal composition, pH-dependent color behavior, antioxidant functionality, and formulation-level performance is necessary to support cleaner production pathways for grape pomace-derived colorants.

In parallel with these developments, recent trends in cosmetic formulation have increasingly explored the direct incorporation of solid plant-based matrices into creams and personal care products, motivated by consumer demand for natural, minimally processed, and visibly botanical ingredients. While such approaches may be suitable for specific applications, such as exfoliating or rinse-off products, their use in leave-on formulations requires careful evaluation. The presence of solid residues can negatively affect formulation homogeneity, optical behavior, sensory perception, and batch-to-batch reproducibility, particularly when color performance and skin feel must be precisely controlled. Consequently, the direct use of agro-industrial residues may limit the functional expression of bioactive compounds such as anthocyanins, underscoring the need for extraction-based strategies that enhance their release and bioavailability, controlled chromatic expression, and biocompatibility with cosmetic matrices. Based on the known variability in anthocyanin composition among grape cultivars, we hypothesize that varietal differences significantly influence chromatic stability and functional performance of grape pomace extracts under near-physiological pH conditions (4.5–5.5), with more structurally complex and acylated anthocyanin profiles leading to improved color stability and formulation compatibility.

In this context, the present study investigates the upcycling of grape pomace from four widely cultivated wine grape varieties (Malbec, Merlot, Syrah, and Grenache) for bio-based cosmetic applications using a two-step strategy. First, the direct incorporation of solid grape pomace residues into cream formulations is evaluated to assess limitations related to color control and homogeneity performance. Subsequently, anthocyanin-rich extracts obtained using bio-based solvents are investigated as soluble, formulation-compatible alternatives, with systematic evaluation of extraction efficiency, varietal effects, pH-responsive color behavior, thermal and photostability, antioxidant performance, and functional behavior in cosmetic creams. This work contributes to cleaner production strategies by providing a rational framework for designing sustainable, variety-specific color-functional ingredients derived from winery residues.

## 2. Materials and Methods

### 2.1. Materials

All chemicals and reagents used in this study were of analytical grade and obtained from Sigma-Aldrich (St. Louis, MO, USA), unless otherwise specified. These included citric acid (≥99%), sulfuric acid (95–98%), sodium hydroxide (≥98%), gallic acid (≥97%), Folin–Ciocalteu reagent, ABTS (2,2′-azinobis(3-ethylbenzothiazoline-6-sulfonic acid), ≥98%), potassium persulfate (≥99%), DPPH (2,2-diphenyl-1-picrylhydrazyl, ≥97%), Trolox (≥97%), ethanol (99.8%), and glacial acetic acid (≥99.7%). Ultrapure water was used for the preparation of all solutions and throughout all experimental procedures.

### 2.2. Raw Material

Grape pomace derived from four *Vitis vinifera* L. wine grape varieties (Malbec, Merlot, Syrah, and Grenache) was obtained from discarded grapes collected at the vineyard level prior to vinification (i.e., not subjected to industrial winemaking processes). The grapes were cultivated in vineyards located in La Palma-Quillota, Region of Valparaiso, Chile (32°50′ S; 71°13′ W; 120 m.a.s.l.). Discarded grape collections were washed, manually destemmed, and mechanically homogenized to remove residual juice. The resulting pomace was therefore produced under controlled laboratory conditions to simulate the solid fraction typically generated during vinification. The material was filtered to eliminate excess liquid and dried at 35 °C for 24 h in thin layers to ensure homogeneous dehydration. Seeds were manually separated, and the dried pomace was milled and sieved to obtain a uniform particle size of 250 µm. The processed material was stored at 25 °C in hermetically sealed containers and protected from light until further use.

### 2.3. Solid–Liquid Extraction of Different Grape Varieties

To evaluate varietal effects, grape pomace from Malbec, Merlot, Syrah, and Grenache was extracted using 50% (*v*/*v*) ethanol–H_2_O pH 2.0 [28]. Extractions were conducted using 0.05 g of pomace (particle size 250 µm) and 10 mL of solvent at 25 °C and 45 °C under constant agitation (500 rpm) for 60 min. The suspension was centrifuged at 2500× *g* for 3 min, and the resulting extracts were subjected to total phenolic content and anthocyanins determination and colorimetric analysis.

### 2.4. Stability of Extracts Under Temperature and Light

After extraction, 5 mL of each varietal extract were transferred into 10 mL glass tubes, sealed to minimize evaporation, and subjected to stability assays. Samples were stored at controlled temperatures of 25 °C and 45 °C using thermostatic incubators. Light exposure was evaluated by storing samples under continuous ambient laboratory light conditions, while dark conditions were ensured by wrapping the tubes in aluminum foil. The combined effects of temperature and light were assessed over a period of 288 h. Total anthocyanin content was determined at 72 h intervals using the pH differential method, and stability was evaluated based on the relative retention of anthocyanins over time. For kinetic analysis, anthocyanin degradation was modeled using a pseudo-first-order approach, assuming ln(C_t_/C_0_) = −kt, where C_t_ is the anthocyanin concentration at time t and C_0_ is the initial concentration. The rate constant (*k*) was obtained from the slope of the linear regression of ln(C_t_/C_0_) versus time. The half-life (t½) was calculated as t½ = ln(0.5)/k [29].

### 2.5. Effect of pH on Anthocyanin Color Appearance

Anthocyanin concentrations of all extracts were standardized to 0.10 mg L^−1^ in order to evaluate the effect of pH on color appearance independently of concentration differences among varieties. Buffer and pH-adjusted solutions covering a range from pH 1 to 14 were prepared by acidification with sulfuric acid or citric acid to obtain acidic conditions, and by alkalinization with sodium hydroxide to achieve basic conditions. Subsequently, 1 mL of each pH-adjusted solution was mixed with 100 µL of the standardized extract, and color parameters were determined using a colorimeter. The pH of the samples was measured using a benchtop pH meter (PHS-W series, Bante Instruments, Shanghai, China). The instrument was calibrated using standard buffer solutions at pH 4.0 and 7.0 prior to analysis. Measurements were performed at room temperature, and all samples were analyzed in triplicate to ensure reproducibility.

### 2.6. Preparation of Cosmetic Cream Formulations

Cosmetic cream formulations were prepared using a commercial cream base (Reachem, Santiago, Chile) following a two-step experimental approach designed to compare the direct use of solid grape pomace residues with anthocyanin-rich extracts as color-functional ingredients. In the first step, solid grape pomace residues from different grape varieties (Malbec, Merlot, Syrah, and Grenache) were dried, finely ground (particle size 100 µm), and directly incorporated into the cream base at two low concentrations (0.1% and 0.5%, *w*/*w*). The formulations were homogenized under mechanical stirring until visual dispersion was achieved. These formulations were prepared to assess the impact of solid residue incorporation on color attributes, homogeneity, and overall formulation appearance. In the second step, anthocyanin-rich extracts dissolved in a 50% (*v*/*v*) ethanol–H_2_O solvent system adjusted to pH 2.0 were incorporated into the same commercial cream base. Two extract loadings (4.5% and 9% (*w*/*w*)) were prepared for each grape variety. The formulations were homogenized until uniform appearance was obtained. For all formulations, pH values were measured at day 0 and after 30 days of storage to assess compatibility with the physiological pH range of human skin and to monitor potential pH changes over time. Color stability was evaluated every two days over a 30-day storage period using a colorimeter, and color parameters were expressed in the CIELab color space.

### 2.7. Analytical Determinations

Total anthocyanin content was determined spectrophotometrically using the pH differential method, as outlined by Lee et al. [30], and the results were expressed in milligrams per liter (mg L^−1^). Briefly, extracts were diluted separately in potassium chloride buffer (0.025 M, pH 1.0) and sodium acetate buffer (0.4 M, pH 4.5). Absorbance was measured at 520 and 700 nm using a 1 cm pathlength cuvette. The corrected absorbance (A) was calculated following Equation (1):

A = (A_520_ − A_700_)pH_1.0_ − (A_520_ − A_700_)pH_4.5_ (1)

Total anthocyanin content was expressed as cyanidin-3-glucoside equivalents (mg L^−1^), using a molecular weight of 449.2 g mol^−1^ and a molar extinction coefficient of 26,900 L mol^−1^ cm^−1^.

Total phenolic content was determined using the Folin–Ciocalteu method as described by Singleton et al. [31]. Briefly, 500 μL of the diluted extract (1:10) were added to a 7.5 mL of distilled water, added 2 mL of Folin-Coicalteu 1 N and after 3 min added 3 mL of Na_2_CO_3_ (10% *v*/*v*), then added water to a final volume of 25 mL with distilled water. The blank was performed with 500 μL of the solvent instead of the extract. Samples were incubated in the dark for 2 h and absorbance was measured at 760 nm. Results were expressed as micrograms of gallic acid equivalents per gram of sample (µg GAE g^−1^).

Antioxidant activity was assessed using ABTS and DPPH radical scavenging assays. The ABTS^+^ assay was conducted according to Arnao [32]. Briefly, 1 mL of the extract was added to a 1 mL of activated ABTS. Samples were incubated in the dark for 7 min and absorbance was measured at 734 nm, and the DPPH assay followed the method of Moreira et al. [33]. Similar to ABTS, DPPH was performed using 1 mL of the extract mixed with 1 mL of DPPH 0.12 mM, incubated for 30 min in the dark and absorbance measured at 517 nm. The blank for both assays was performed with 1 mL of the solvent instead of the extract. Trolox was used as the reference standard, and results were expressed as Trolox equivalents per gram of sample (μmol TE g^−1^).

Color properties of the extracts were evaluated using the CIELab color space. Measurements were performed with an LS173 colorimeter (Linshang Technology Co., Ltd., Shenzhen, China), equipped with a D65 standard illuminant and a 10° standard observer, operating over the visible spectral range (400–700 nm). The instrument measures reflected light and converts spectral data into CIELab coordinates (L*, a*, b*) using internal colorimetric parameters. Prior to measurements, the device was calibrated using a standard white reference plate. Samples were analyzed in “Color Scan” mode by direct contact with the measurement surface to ensure consistent geometry and minimize external light interference. All measurements were performed in triplicate under identical conditions. When applicable, color differences (ΔE*ab) were calculated using the instrument’s internal color comparison function.

### 2.8. Statistical Analysis

Data are expressed as mean ± standard deviation (n ≥ 3). Due to the small sample size and non-normal data distribution, statistical differences among groups were assessed using the Kruskal–Wallis test followed by Dunn’s post hoc test. Exact *p*-values were calculated where applicable, and significance was set at *p* < 0.05. Analyses were performed using GraphPad Prism 9.0 (GraphPad Software, San Diego, CA, USA).

## 3. Results and Discussion

### 3.1. Direct Incorporation of Grape Pomace Residues into Cosmetic Creams: Color Performance and Formulation Limitations

**Figure 1:**
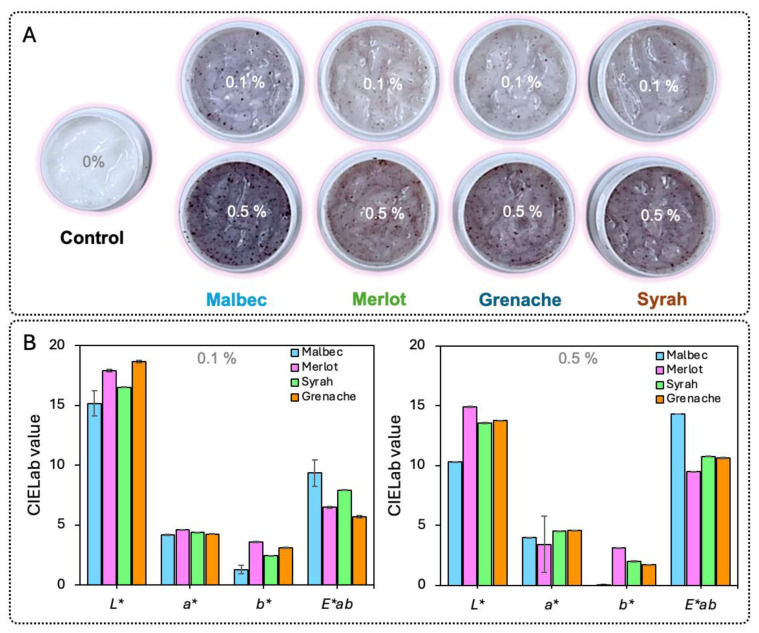
Visual appearance and CIELab color parameters of cosmetic creams containing solid grape pomace residues. (**A**) Cream formulations without residue (control) and with grape pomace residues from different grape varieties (Malbec, Merlot, Grenache, and Syrah) incorporated at 0.1% and 0.5% (*w*/*w*). (**B**) CIELab values of the corresponding formulations at 0.1% and 0.5% residue loading.

The direct incorporation of solid grape pomace residues from different grape varieties (Malbec, Merlot, Syrah, and Grenache) into cosmetic cream formulations was evaluated at two low incorporation levels, 0.1% and 0.5% (*w*/*w*), in order to assess their impact on color attributes and formulation homogeneity. Color parameters were measured using the CIELab color space (L*, a*, b*, and ΔE*ab) (Figure 1).

At a residue loading of 0.5%, all formulations exhibited a pronounced reduction in lightness (L*), indicating significant darkening of the cream matrix. L* values ranged from 10.32 for Malbec to 14.89 for Merlot, with Syrah and Grenache showing intermediate values of 13.56 and 13.75, respectively. These low L* values reflect the strong optical density introduced by the solid residues. The red–green coordinate (a*) was positive for all varieties, ranging from 3.40 (Merlot) to 4.56 (Grenache), indicating a shift toward red hues. However, the yellow–blue coordinate (b*) remained low, particularly for Malbec (b* = 0.05), suggesting limited chromatic definition and a tendency toward dark, muted coloration rather than a clear red tone. As a consequence, overall color differences (ΔE*ab) were high for all formulations, reaching 14.30 for Malbec, 10.77 for Syrah, 10.63 for Grenache, and 9.48 for Merlot, values that correspond to clearly perceptible and visually dominant color changes. Reducing the residue incorporation level to 0.1% led to an increase in L* values for all grape varieties, indicating lighter formulations. At this lower loading, L* values increased to 15.15 (Malbec), 17.88 (Merlot), 16.51 (Syrah), and 18.63 (Grenache). Despite this increase in lightness, ΔE*ab values remained elevated, ranging from 5.67 for Grenache to 9.34 for Malbec. These values are still above the commonly accepted perceptibility threshold for cosmetic products (ΔE*ab ≈ 3), indicating that color differences would remain clearly noticeable to consumers even at the lowest tested residue concentration. The a* coordinate remained relatively stable across concentrations, with values between 4.16 and 4.60, while b* values increased slightly compared to the 0.5% formulations, suggesting a modest contribution of yellow tones as the influence of the cream base became more pronounced. Across both concentrations, Malbec residues consistently produced the strongest chromatic impact, exhibiting the lowest L* values and the highest ΔE*ab values. This behavior is consistent with the higher anthocyanin content typically reported for Malbec grape skins (discussed in the next section). However, even varieties with lower pigment content, such as Merlot and Grenache, produced ΔE*ab values between 5.67 and 9.48, confirming that the intense color impact is not exclusive to a single variety but intrinsic to the use of solid residues.

The pronounced color changes observed for all formulations can be attributed primarily to physical effects associated with the presence of insoluble particles, rather than to controlled color expression. Solid residues composed of fibrous skin material and residual seed fragments promote heterogeneous light absorption and scattering within the cream matrix, leading to opaque, poorly defined coloration. This physical mechanism explains why reducing the residue concentration from 0.5% to 0.1% was insufficient to bring ΔE*ab values into an acceptable range for cosmetic applications. In addition to chromatic effects, the direct use of solid grape pomace residues negatively affected formulation homogeneity and sensory performance. Visual inspection revealed non-uniform dispersion of particles throughout the cream, and after topical application, residual particulates remained visible on the skin surface (Figure 1A). Such characteristics are incompatible with leave-on cosmetic products, where smooth texture, uniform appearance, and absence of perceptible solids are essential for consumer acceptance. These results demonstrate that although grape pomace residues contain valuable pigmented compounds, their direct incorporation into cosmetic creams results in intense, poorly controlled, and heterogeneous color effects that cannot be adequately mitigated by simple dilution. Consequently, the isolation of anthocyanins through extraction is a necessary step to eliminate the solid matrix responsible for these physical interferences, enabling the development of homogeneous, controllable, and cosmetically acceptable color systems. This finding provides the technological rationale for the extraction-based approach evaluated in the subsequent sections of this study.

### 3.2. Varietal Influence on Colored Extract Performance

**Figure 2:**
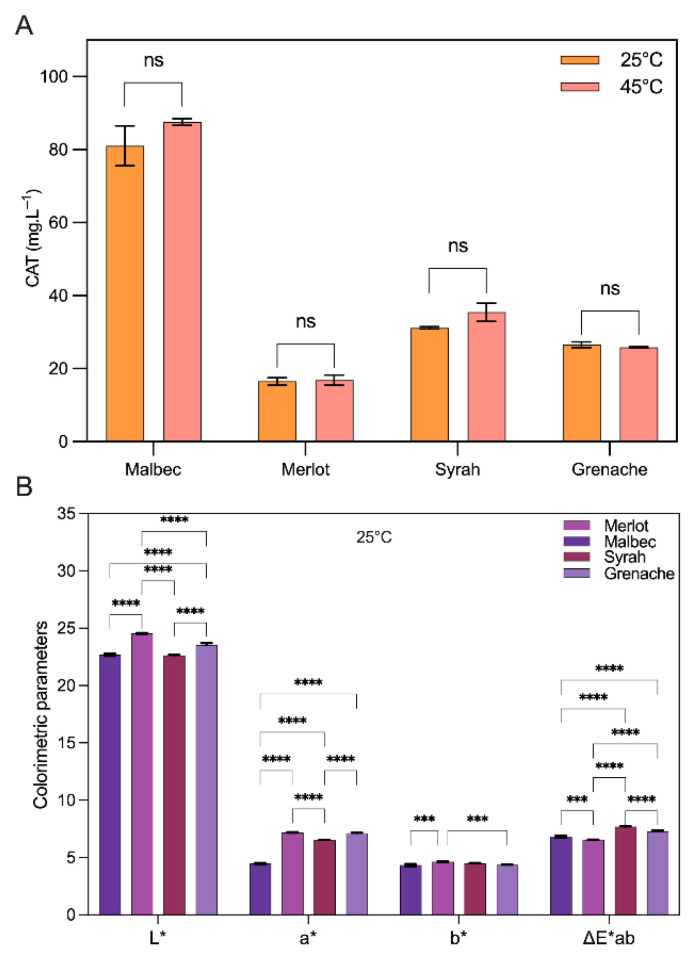
Effect of grape variety on anthocyanin recovery and color properties of grape pomace extracts obtained using 50% (*v*/*v*) ethanol–H_2_O at pH 2.0. (**A**) Total anthocyanin content of Malbec, Merlot, Syrah, and Grenache extracts obtained at 25 and 45 °C, showing no significant differences between temperatures within each variety (*p* > 0.05). (**B**) CIELab color parameters (L*, a*, b*) and overall color difference (ΔE*ab) of varietal extracts measured at 25 °C. Statistical significance is indicated as follows: ns, not significant (*p* > 0.05); *** *p* < 0.001; **** *p* < 0.0001.

Based on the previous work [28], 50% (*v*/*v*) ethanol–H_2_O acidified to pH 2.0 was selected as the optimal extraction medium, as it provided the highest anthocyanin yield while maintaining acceptable stability under different storage conditions. By fixing the extraction solvent and pH, the subsequent analysis focuses on isolating the effect of grape variety on extract composition and color-related properties. In this context, Figure 2 compares the anthocyanin content and associated parameters of extracts obtained from Malbec, Merlot, Syrah, and Grenache grape pomace under standardized extraction conditions.

Figure 2A illustrates the effect of grape variety and extraction temperature on anthocyanin recovery from grape pomace using 50% (*v*/*v*) ethanol–H_2_O at pH 2.0. It should be noted that the absolute anthocyanin concentrations obtained in this study are inherently dependent on factors such as grape variety, vintage, agronomic conditions, and pomace composition, and therefore should be interpreted in a comparative rather than absolute sense. Under these conditions, and at 25 °C, the extraction yield was strongly dependent on the grape variety, with Malbec (used in this work as the reference matrix) exhibiting the highest anthocyanin content (81.05 mg L^−1^), followed by Syrah (31.19 mg L^−1^), Grenache (26.55 mg L^−1^), and Merlot (16.50 mg L^−1^). Although slightly higher anthocyanin values were observed at 45 °C for Malbec and Syrah, no statistically significant differences were detected between extractions conducted at 25 and 45 °C for any of the evaluated varieties (*p* > 0.05). This indicates that, within the tested temperature range, the system operates in a regime where mass transfer limitations are not substantially alleviated by moderate heating, or where gains in diffusional transport are counterbalanced by concurrent thermal degradation or pigment transformation. These findings indicate that increasing the extraction temperature from 25 to 45 °C does not provide a meaningful process advantage in terms of anthocyanin recovery under the selected operating conditions. This observation is consistent with previous studies reporting a negligible effect of temperature within the 25–45 °C range on anthocyanin extraction from grape pomace using acidified ethanol–water systems. For instance, Ju and Howard [34], reported antioxidant capacity (ORAC) values of approximately 2772 and 2967 µmol TE g^−1^ at 20 °C and 40 °C, respectively, with no statistically significant differences between temperatures. Consequently, lower-temperature operation may be preferred from an energy-efficiency and pigment-preservation perspective, particularly in the context of sustainable bioprocess design and scale-up of anthocyanin-rich extracts from grape pomace.

Figure 2B shows the CIELab color parameters of grape pomace extracts obtained at 25 °C, which can be directly interpreted in light of the anthocyanin concentrations reported in Figure 2A. In general, an inverse relationship between pigment concentration and lightness (L*) was observed, where extracts with higher anthocyanin content exhibited darker color intensity. Malbec, which showed the highest anthocyanin concentration at 25 °C (81.05 mg L^−1^), presented one of the lowest L* values (22.69), whereas Merlot, with the lowest anthocyanin concentration (16.50 mg L^−1^), exhibited the highest L* value (24.55). Syrah (31.19 mg L^−1^) and Grenache (26.55 mg L^−1^) displayed intermediate L* values of 22.62 and 23.53, respectively, confirming that increased pigment loading reduces extract lightness due to higher optical density. The red–green coordinate (a*) did not scale linearly with total anthocyanin concentration. Although Malbec had the highest pigment content, it showed the lowest a* value (4.46), while Merlot and Grenache, despite lower anthocyanin levels, exhibited higher a* values of 7.19 and 7.12, respectively. Syrah presented an intermediate a* value (6.51). This behavior indicates that chromatic expression along the red axis is governed not only by pigment concentration but also by varietal-dependent anthocyanin profiles. The b* coordinate remained relatively constant across all varieties, ranging from 4.34 to 4.63, indicating that yellow–blue contributions were minimally affected by differences in anthocyanin concentration or grape variety under the selected process conditions. As a result, the overall color difference (ΔE*ab) among extracts reflected combined effects of pigment concentration and qualitative color expression. Syrah and Grenache exhibited the highest ΔE*ab values (7.69 and 7.27, respectively), followed by Malbec (6.83) and Merlot (6.55), indicating perceptible varietal color differences even under identical extraction conditions.

### 3.3. pH-Dependent Color Stability and Chromatic Response

**Figure 3:**
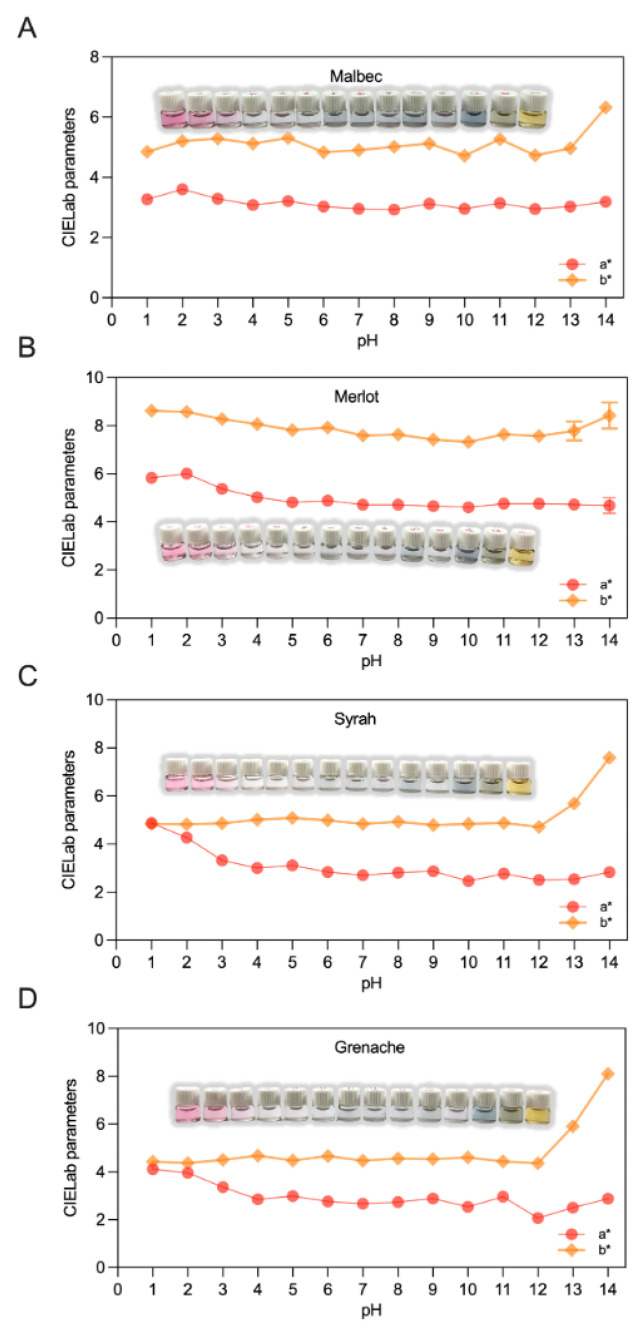
pH-dependent color stability of standardized anthocyanin extracts from (**A**) Malbec, (**B**) Merlot, (**C**) Syrah, and (**D**) Grenache grape pomace, expressed by CIELab parameters (L*, a*, b*) across a pH range of 1–14.

To isolate the effect of pH on color stability, anthocyanin concentrations were standardized for all extracts prior to analysis. This normalization eliminates concentration-dependent optical effects, enabling direct comparison of pH-induced color changes among grape varieties. Consequently, the observed differences in color behavior reflect intrinsic varietal anthocyanin composition and stability rather than differences in pigment loading (Figure 3). Anthocyanin composition patterns for *Vitis vinifera* cultivars are well established in the literature and provide a reliable basis for mechanistic interpretation [35,36,37]. These studies indicate that varietal differences are primarily associated with the relative abundance and structural diversity of anthocyanins, particularly delphinidin-, cyanidin-, petunidin-, peonidin-, and malvidin-3-*O*-glucosides, as well as their acylated derivatives. Malbec is typically characterized by a more complex anthocyanin profile, comprising high levels of malvidin-3-*O*-glucoside together with significant contributions from delphinidin- and petunidin-based glucosides, as well as acylated derivatives such as malvidin-3-*O*-(6-O-acetyl)-glucoside and malvidin-3-*O*-(6-*O*-p-coumaryl)-glucoside. These acylated anthocyanins enhance color stability through intramolecular copigmentation and increased resistance to hydration reactions. In contrast, Merlot is generally dominated by malvidin-3-*O*-glucoside and peonidin-3-*O*-glucoside, with a comparatively lower proportion of acylated forms. Syrah presents a profile rich in malvidin-3-*O*-glucoside, peonidin-3-*O*-glucoside, and acylated anthocyanins, including both acetylated and p-coumaroylated derivatives, which contribute to enhanced color intensity and stabilization. Grenache, on the other hand, typically shows a simpler anthocyanin composition, mainly consisting of malvidin-3-*O*-glucoside and cyanidin-3-*O*-glucoside, with lower total anthocyanin content and fewer acylated species [35,36,37].

Figure 3 shows that pH strongly affects the color stability and chromatic response of anthocyanin extracts, with clear variety-dependent behavior across the pH range from 1 to 14. For all grape varieties, color variations reflect well-established pH-driven structural transformations of anthocyanins, transitioning from the red flavylium cation under acidic conditions to quinoidal bases at intermediate pH, and to chalcone and degradation products under alkaline conditions. In Malbec extracts (Figure 3A), the a* coordinate showed a gradual decrease from 3.60 at pH 2 to 2.96–3.03 between pH 7 and 13, indicating partial loss of red chromaticity with increasing pH. At pH 14, a marked increase in b* (6.32) suggests the formation of yellowish degradation products. Merlot extracts (Figure 3B) exhibited a decline in a* from 6.00 at pH 2 to approximately 4.60–4.75 between pH 7 and 14, accompanied by consistently high b* values (7.32–8.62), indicating a persistent yellow contribution. Syrah extracts (Figure 3C) showed a more pronounced decrease in a* (from 4.87 at pH 1 to 2.46 at pH 10), reflecting higher sensitivity of red chromaticity to pH changes, while b* increased significantly under alkaline conditions. Similarly, Grenache extracts (Figure 3D) exhibited a strong decrease in a* (from 4.11 at pH 1 to 2.07 at pH 12) and a sharp increase in b* at pH 13–14, indicating advanced pigment transformation and degradation.

The more pronounced decrease in a* observed for Syrah, Grenache, and Merlot extracts indicates a higher sensitivity of red chromaticity to pH changes compared to Malbec. This behavior is consistent with a faster loss of the flavylium cation form and a greater tendency toward structural transformations into less colored species as pH increases. Such differences can be associated with varietal anthocyanin composition, including the relative abundance of malvidin-, delphinidin-, and cyanidin-based glucosides, as well as the extent of acylation and copigmentation interactions [35,36,37]. These patterns are in agreement with the established pH-dependent equilibria of anthocyanins, where acidic conditions (pH < 3) favor the red flavylium cation, intermediate pH values promote the formation of less stable quinoidal and carbinol forms, and alkaline conditions induce ring opening and the formation of yellowish chalcone structures. Within this framework, varietal differences can be interpreted in terms of anthocyanin structural features. In particular, acylated anthocyanins, reported in higher proportions in certain cultivars, can enhance color stability through intramolecular copigmentation, whereas simpler anthocyanin profiles with fewer stabilizing substituents are more susceptible to hydration and degradation reactions at elevated pH. The experimental results obtained in this study are consistent with this interpretation. The greater chromatic stability of Malbec extracts under acidic and near-neutral conditions suggests a more favorable balance of structurally diverse and potentially more stabilized anthocyanins, while the sharper decrease in a* observed for Syrah, Grenache, and Merlot reflects a higher susceptibility of their dominant anthocyanin forms to pH-induced transformations.

In practical terms, these findings demonstrate that grape variety defines the operational pH window for color stability when concentration effects are controlled. Malbec exhibited the highest chromatic robustness under acidic and near-neutral conditions, whereas Syrah, Grenache, and Merlot showed greater sensitivity to pH-induced color loss. This behavior is particularly relevant for cosmetic cream formulations, where maintaining color stability at near-physiological pH is essential for product performance and consumer acceptance.

### 3.4. Total Phenolic Content and Antioxidant Activity

**Figure 4:**
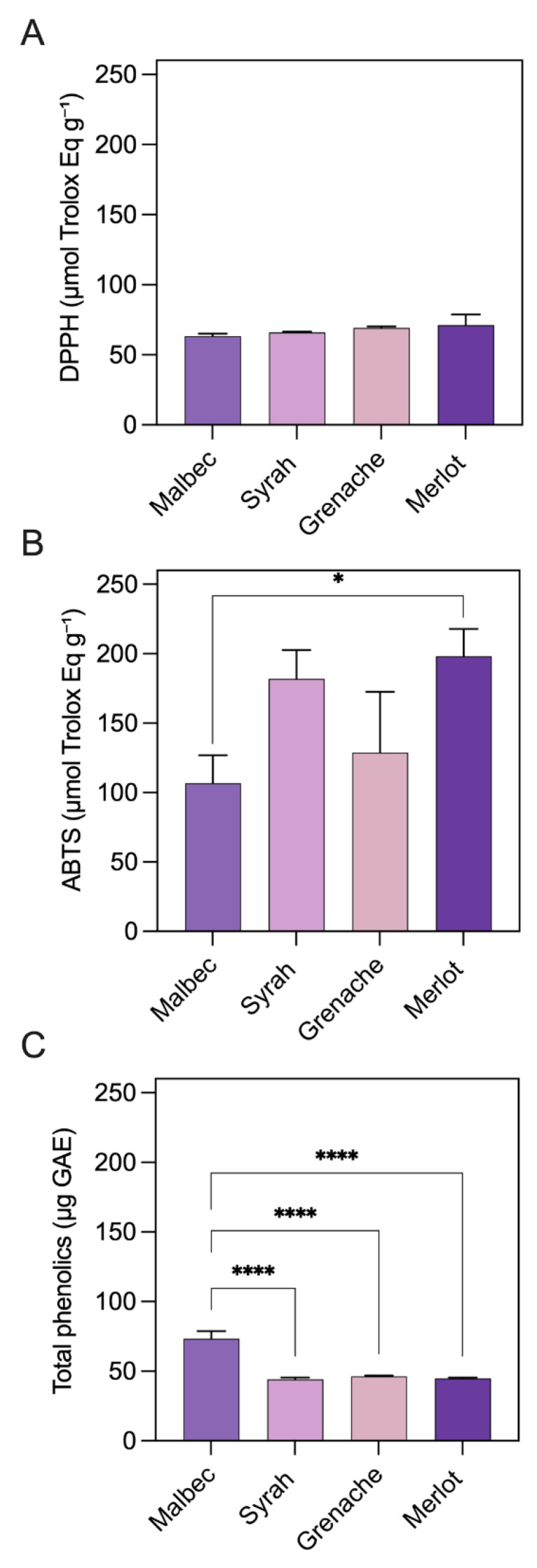
Antioxidant activity and total phenolic content of grape pomace extracts. (**A**) DPPH radical scavenging activity expressed as μmol TE g^−1^. (**B**) ABTS radical scavenging activity expressed as μmol TE g^−1^. (**C**) Total phenolic content expressed as µg GAE g^−1^. Asterisks indicate statistically significant differences among grape varieties (*p* < 0.05).

Beyond color performance, anthocyanin-rich extracts intended for cosmetic applications must also exhibit biological functionality, particularly antioxidant activity, which contributes to product stability and potential skin-protective effects. In this context, the antioxidant capacity of the grape pomace extracts was evaluated through total phenolic content determination and radical scavenging assays (ABTS and DPPH). These analyses provide complementary insight into the functional quality of the extracts and support their suitability for incorporation into color-critical cosmetic formulations. Figure 4 summarizes the antioxidant performance and total phenolic content of grape pomace extracts. As shown in Figure 4A, DPPH radical scavenging activity, expressed as Trolox equivalents, ranged from 63.37 to 71.18 μmol TE g^−1^. Although Merlot exhibited the highest DPPH value (71.18 μmol TE g^−1^), no statistically significant differences were detected among varieties (*p* > 0.05), indicating comparable hydrogen-donating capacity across the extracts. In contrast, ABTS radical scavenging activity (Figure 4B) showed significant varietal differences. Merlot and Syrah exhibited the highest antioxidant capacities, reaching 198.09 and 181.96 μmol TE g^−1^, respectively, which were significantly higher than Malbec (106.65 μmol TE g^−1^), as indicated by the statistical grouping (*p* < 0.05). Grenache displayed intermediate ABTS activity (141.35 μmol TE g^−1^), reflecting a moderate electron-transfer capacity relative to the other varieties. Total phenolic content (Figure 4C) differed markedly among the extracts. Malbec presented a significantly higher phenolic concentration (73.25 µg GAE g^−1^) compared to Syrah (44.01 µg GAE g^−1^), Grenache (46.17 µg GAE g^−1^), and Merlot (44.65 µg GAE g^−1^). These results confirm that Malbec pomace is particularly rich in phenolic compounds, although this higher phenolic load did not translate into the highest antioxidant activity.

Notably, TPC exhibits poor correlation with radical scavenging capacities (DPPH/ABTS) across grape pomace extracts, attributable to heterogeneous phenolic compositions comprising flavan-3-ols, phenolic acids, and anthocyanin subtypes beyond bulk GAE equivalence [38]. For example, Malbec’s highest TPC (73.25 µg GAE g^−1^) reflects its elevated flavan-3-ol (catechin, epicatechin) and phenolic acid content in pomace, compensating for modest anthocyanins, yet yielding lower ABTS (106.65 μmol TE g^−1^) because these compounds favor hydrogen atom transfer (DPPH) over single electron transfer (ABTS). Merlot and Syrah’s superior ABTS (198.09 and 181.96 μmol TE g^−1^) stems from acylated anthocyanins and higher procyanidin oligomers, which excel in single electronic transfer mechanisms despite lower TPC (~44–46 µg GAE g^−1^), explaining the lack of direct TPC-antioxidant correlation (common in grape byproducts) [38,39].

### 3.5. Thermal and Photostability

**Figure 5:**
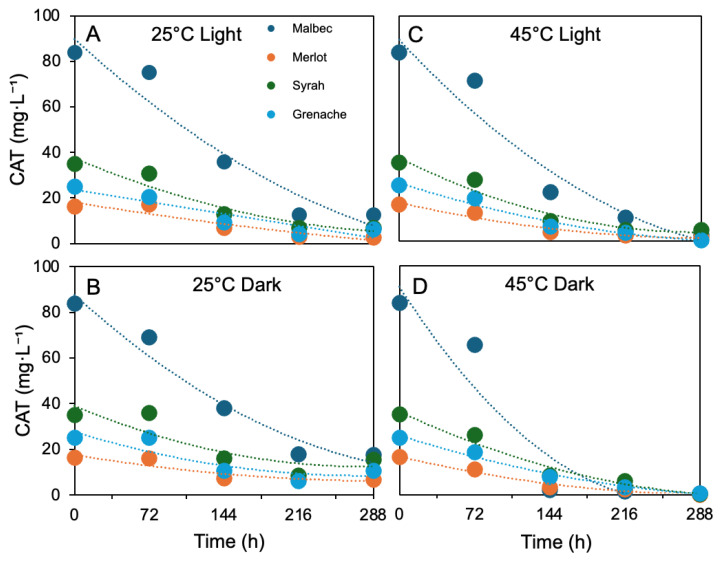
Thermal and photostability of grape pomace anthocyanin extracts in the selected 50% (*v*/*v*) ethanol–H_2_O pH 2.0. Total anthocyanin content was monitored over 288 h under different storage conditions: at 25 °C in the presence (**A**) and absence (**B**) of light, and at 45 °C in the presence (**C**) and absence (**D**) of light.

The stability of anthocyanin-rich grape pomace extracts was evaluated in the selected 50% (*v*/*v*) ethanol–H_2_O pH 2.0, under controlled temperature (25 and 45 °C) and light conditions over 288 h. Even under acidic conditions, which favor the flavylium cation and are generally associated with enhanced anthocyanin stability, pronounced degradation trends were observed, strongly controlled by temperature, light exposure, and grape variety (Figure 5).

As depicted in Figure 5A, at 25 °C under light exposure, all extracts showed progressive anthocyanin loss. Malbec decreased from 83.74 to 12.59 mg L^−1^ after 288 h, while Syrah and Grenache reached 6.87 and 6.31 mg L^−1^, respectively. Merlot showed the lowest residual anthocyanin content (2.46 mg L^−1^). When stored at 25 °C in the absence of light (Figure 5B), degradation was significantly reduced, with Malbec and Syrah retaining 17.59 and 15.58 mg L^−1^ after 288 h, respectively, confirming the protective effect of light exclusion even at pH 2.0. At 45 °C, anthocyanin degradation was markedly accelerated despite the acidic environment. Under light exposure (Figure 5C), Malbec retained only 2.85 mg L^−1^ after 288 h, while Grenache and Merlot showed near-complete pigment loss. Storage at 45 °C in the dark resulted in even lower residual anthocyanin levels, with Malbec decreasing to 0.50 mg L^−1^ and Merlot showing complete degradation, indicating that thermal degradation dominated pigment stability under these conditions.

Anthocyanin degradation in grape pomace extracts followed pseudo-first-order kinetics, *viz*., both temperature, light exposure, and grape variety influenced stability. At 25 °C under light, k values ranged from 6.02 to 7.74 × 10^−3^ h^−1^, with Grenache showing the lowest degradation rate and longest half-life (115.14 h), indicating higher stability, while Malbec and Merlot degraded slightly faster. Under dark conditions at 25 °C, degradation slowed further for all varieties, particularly Merlot (k = 3.68 × 10^−3^ h^−1^; t½ = 188.16 h), followed by Syrah and Grenache, confirming improved stability without light. At 45 °C, degradation accelerated in all varieties. Under light, Malbec and Grenache showed higher k values (1.21–1.28 × 10^−2^ h^−1^), whereas Merlot remained comparatively more stable (k = 8.56 × 10^−3^ h^−1^). Under dark conditions at 45 °C, the highest degradation rates were observed for Malbec and Syrah (1.96 × 10^−2^ and 1.64 × 10^−2^ h^−1^, respectively), while Merlot and Grenache showed lower rates, indicating better thermal resistance. Overall, Malbec and Syrah were more sensitive to thermal stress, whereas Merlot and Grenache generally exhibited higher stability depending on storage conditions. The *k* values obtained in this study are in good agreement with those reported by Harbourne et al. [40] for model systems, confirming pseudo-first-order kinetics and the high thermal sensitivity of anthocyanins. Consistently, both studies show an increase in *k* with temperature and a corresponding decrease in half-life (t½), highlighting the thermal instability of these compounds. However, the present work extends these findings by demonstrating, in a real grape pomace matrix, the additional influence of light exposure and varietal differences on degradation kinetics. These results demonstrate that although acidic conditions (pH 2.0) promote anthocyanin stabilization, temperature remains the primary limiting factor governing pigment preservation during storage [9,41]. In fact, maintaining moderate temperatures and minimizing light exposure are critical to preserving anthocyanin content in ethanol–H_2_O systems, particularly for applications requiring extended shelf life.

### 3.6. Functional Performance of Extracts in Topical Formulations

**Figure 6:**
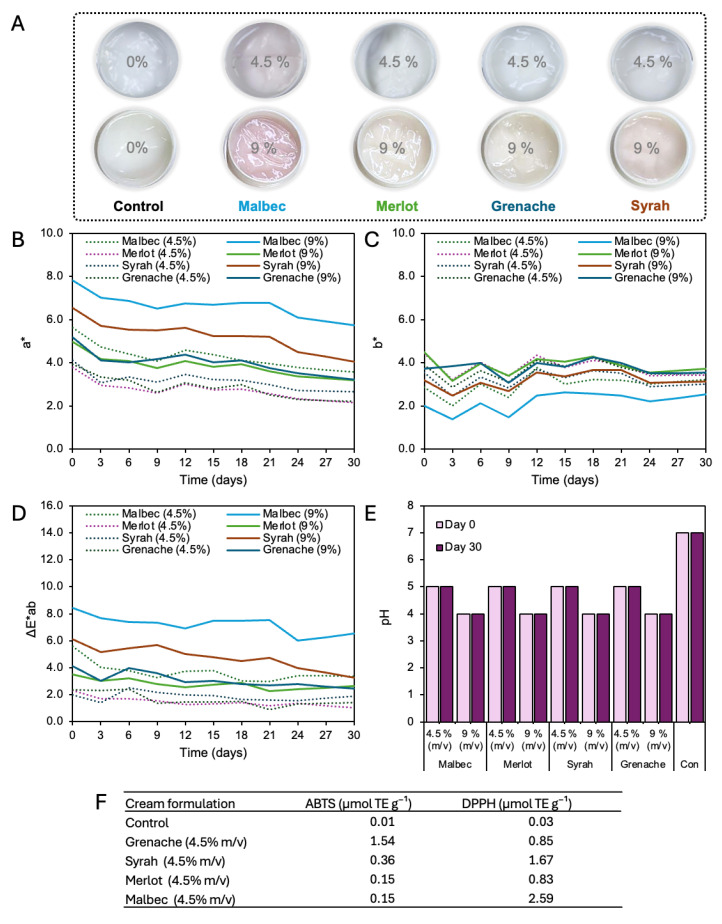
Color, pH, and antioxidant properties of cream formulations containing grape pomace extracts. (**A**) Visual appearance at 4.5 and 9% (m/v); (**B**–**D**) color parameters (a*, b*, ΔE*ab); (**E**) pH values; and (**F**) ABTS and DPPH antioxidant activity (4.5% m/v).

The color stability of cosmetic cream formulations containing grape pomace extracts was strongly influenced by both extract concentration and grape variety over the 30-day storage period (Figure 6A). For all varieties, increasing extract concentration from 4.5% to 9% (m/v) resulted in higher initial a* values (Figure 6B), indicating enhanced red chromaticity, followed by a gradual decrease over time for all varieties. Malbec-based creams exhibited the most intense red coloration, particularly at 9% (m/v), with a decreasing from 7.82 at day 0 to 5.74 at day 30. Although a reduction in red intensity was observed, overall color differences (ΔE*ab) remained relatively stable after the initial storage period, decreasing from approximately 8.44 to 6.51. At 4.5% (m/v), Malbec formulations showed lower ΔE*ab values (≈3–4 after day 6), indicating moderate but acceptable color changes. ΔE*ab values below 3 are generally associated with low perceptibility by the human eye, while values above 3 indicate more visible color differences. This criterion is widely applied in cosmetic and other formulation studies to evaluate color acceptability [42]. Merlot formulations demonstrated the highest chromatic stability among all varieties. At 4.5% (m/v), ΔE*ab values remained below 2.3 throughout storage (Figure 6D), reaching approximately 1.03 at day 30, which corresponds to hardly perceptible color differences. Even at 9% (m/v), Merlot creams maintained relatively low ΔE*ab values (2.26–3.48), confirming excellent color stability over time. Syrah-based creams showed intermediate behavior. At 4.5% (m/v), ΔE*ab values remained below 2.5 during storage. However, at 9% (m/v), higher initial ΔE*ab values were observed (≈6.12 at day 0), followed by a progressive decrease to 3.26 at day 30, indicating noticeable but stabilizing color changes. Grenache formulations exhibited moderate stability. At 4.5% (m/v), ΔE*ab values decreased from 2.34 at day 0 to approximately 1.40 at day 30. At 9% (m/v), ΔE*ab values ranged from 4.12 to 2.44, suggesting perceptible but acceptable color variations for cosmetic applications.

Note that, the incorporation of grape pomace anthocyanin extracts into cosmetic cream formulations at 4.5% and 9% (m/v) did not induce changes in product pH over the 30-day storage period (Figure 6E). For all varieties (Malbec, Merlot, Syrah, and Grenache), formulations containing 4.5% extract maintained a pH of 5, while those containing 9% extract stabilized at pH 4 from day 0 to day 30. In contrast, the control formulation without extract remained at pH 7 throughout storage. These results indicate that the extracts are chemically compatible with the cream matrix and do not promote pH drift over time, even at higher loading levels. The final pH values of the 4.5% extract-containing formulations fall within or close to the physiological pH range of human skin (pH 4.5–5.5), indicating their suitability for topical cosmetic applications, which is critical for cosmetic safety, stability, and consumer acceptance. At this concentration, all extract-containing creams exhibited measurable antioxidant activity compared to the control (Figure 6F). Among the varieties, the Malbec-based formulation showed the most favorable performance, combining enhanced chromatic stability with the highest DPPH scavenging activity (2.59 µmol Trolox Eq g^−1^). These results support the selection of Malbec extract at 4.5% (m/v) as the most suitable candidate for cosmetic applications where both color performance and bioactivity are required.

This study demonstrates that grape pomace, a major agro-industrial by-product, can be effectively upcycled into functional colorant ingredients for cosmetic formulations, supporting circular economy strategies and waste valorization. However, the results clearly show that extract performance is not generic across grape pomace sources. Grape variety plays a decisive role in determining color stability, pH responsiveness, and antioxidant functionality, both in solution and in formulated products. Therefore, varietal selection and systematic physicochemical characterization are essential steps prior to application, as extract concentration, stability window, and functional performance vary significantly among varieties. From an industrial perspective, the scalability of anthocyanin extraction is closely associated with the use of cost-effective and environmentally compatible solvents, such as hydroalcoholic or acidified aqueous systems, which are aligned with regulatory requirements for cosmetic applications. Nevertheless, regulatory aspects, including ingredient safety, compositional consistency, and stability, must be carefully addressed to ensure compliance and product reliability. In addition, inter-annual variability in grape pomace composition, influenced by climatic conditions and vintage, may affect anthocyanin profiles and, consequently, color performance and reproducibility. These findings highlight the need for integrated and variety-specific evaluation when developing sustainable, bio-based color systems, ensuring predictable performance, product stability, and successful industrial implementation.

Finally, it is important to note that the grape pomace used in this study was obtained from grapes prior to industrial vinification. In contrast, grape pomace generated during winemaking processes typically undergoes partial extraction of anthocyanins and other phenolics into the wine matrix, as well as oxidative and enzymatic transformations during fermentation. As a result, industrial pomace generally presents lower total anthocyanin content and a modified phenolic profile compared to the material used in this work. Therefore, the anthocyanin yields and functional properties reported here should be interpreted as representative of a high-potential raw material, and caution should be exercised when directly comparing these results with studies based on winery-derived pomace.

## 4. Conclusions

This study demonstrates that grape pomace valorization into anthocyanin-rich extracts is strongly dependent on grape variety and cannot be treated as a generic process. Grape variety plays a decisive role in determining extraction efficiency, color stability, pH responsiveness, and antioxidant functionality, both in solution and in cosmetic formulations. The results also highlight that direct incorporation of solid pomace presents important limitations related to heterogeneity, uncontrolled color expression, and sensory performance, whereas extraction enables improved stability, homogeneity, and functional integration into formulations. Overall, these findings indicate that varietal selection and extraction strategy are key parameters for the development of sustainable, bio-based colorants from winery residues. However, as this study is based on a single harvest and geographical origin, further investigations considering different vintages and growing conditions are required to confirm the broader applicability of these results.

## Data Availability

The original contributions presented in this study are included in the article. Further inquiries can be directed to the corresponding author.
